# Progranulin Mutations Affects Brain Oscillatory Activity in Fronto-Temporal Dementia

**DOI:** 10.3389/fnagi.2016.00035

**Published:** 2016-02-29

**Authors:** Davide V. Moretti, Luisa Benussi, Silvia Fostinelli, Miriam Ciani, Giuliano Binetti, Roberta Ghidoni

**Affiliations:** ^1^Alzheimer Rehabilitation Research Unit, IRCCS Istituto Centro San Giovanni di Dio FatebenefratelliBrescia, Italy; ^2^Molecular Markers Laboratory, IRCCS Istituto Centro San Giovanni di Dio FatebenefratelliBrescia, Italy; ^3^Memory Clinic, IRCCS Istituto Centro San Giovanni di Dio FatebenefratelliBrescia, Italy

**Keywords:** EEG, FTD, PGRN positive, PGRN negative, MCI FTD

## Abstract

**Background:** Mild cognitive impairment (MCI) is a clinical stage indicating a prodromal phase of dementia. This practical concept could be used also for fronto-temporal dementia (FTD). Progranulin (PGRN) has been recently recognized as a useful diagnostic biomarker for fronto-temporal lobe degeneration (FTLD) due to *GRN* null mutations. Electroencephalography (EEG) is a reliable tool in detecting brain networks changes. The working hypothesis of the present study is that EEG oscillations could detect different modifications among FTLD stages (FTD-MCI versus overt FTD) as well as differences between *GRN* mutation carriers versus non-carriers in patients with overt FTD.

**Materials and Methods:** EEG in all patients and PGRN dosage in patients with a clear FTD were detected. The cognitive state has been investigated through mini mental state examination (MMSE).

**Results:** MCI-FTD showed a significant lower spectral power in both alpha and theta oscillations as compared to overt FTD. *GRN* mutations carriers affected by FTLD show an increase in high alpha and decrease in theta oscillations as compared to non-carriers.

**Conclusion:** EEG frequency rhythms are sensible to different stage of FTD and could detect changes in brain oscillatory activity affected by *GRN* mutations.

## Introduction

Fronto-temporal lobar degeneration (FTLD) is a neurodegenerative disorder characterized by behavioral abnormalities, language impairment, and deficits in executive functions as the most typical clinical features (Seelaar et al., 2011). FTLD is clinically heterogeneous, as different clinical variants have been carefully described. On the basis of presenting clinical symptoms, behavioural variant FTD (bvFTD), agrammatic variant of Primary Progressive Aphasia (avPPA), and semantic variant of PPA (svPPA) represent the most common phenotypes (Benussi et al., 2015). Each one presents specific neuroimaging hallmarks; bvFTD is characterized by mesial and dorsolateral frontal damage, prevalent on the right side, avPPA is defined by involvement of Broca’s area and left insula, whilst svPPA usually presents left rostral temporal involvement (Lashley et al., 2015).

Moreover, FTD is chacterized also by early stages, usually named mild cognitive impairment (MCI), that are still not completely characterized. Unfortunately, the diagnosis of FTD can be difficult because of its insidious and gradual onset and can also be misdiagnosed as Alzheimer’s disease (AD) (Rankin et al., 2005; Walker et al., 2005).

The discovery of mutations in *GRN* as a genetic determinant for FTLD resulted in the rapid identification of a large number of families carrying *GRN* mutations, inherited in an autosomal dominant pattern. At present, 77 different mutations in more than 240 unrelated families have been described (Ghidoni et al., 2012a). Mutations in *GRN* have been associated with a broad spectrum of clinical phenotypic variability (Benussi et al., 2008; Rohrer et al., 2010a,b).

GRN null mutations cause protein haploinsufficiency, leading to a significant decrease in progranulin (PGRN) levels that can be detected in plasma, serum, and cerebrospinal fluid of mutation carriers (Ghidoni et al., 2008, 2012b; Finch et al., 2009; Sleegers et al., 2009). It has been reported that PGRN promotes neuronal survival and neurite outgrowth in cultured neurons (Van Damme et al., 2008) and enhances neuronal survival under stress conditions (Kessenbrock et al., 2008). Data from GRN knockout experimental models suggest that PGRN deficiency leads to reduced synaptic connectivity and impaired plasticity, which may be contributing factors to FTLD pathology in human patients (Tapia et al., 2011; Petkau et al., 2012). Brain activity could be widely and variously affected in FTLD patients with *GRN* mutations. Neuroimaging studies have shown that the topography of brain atrophy is frequently asymmetric, and predominantly involves the frontal, temporal, and parietal cortex (Whitwell et al., 2012). Nevertheless, a recent study (Caroppo et al., 2014) have demonstrated that a diffuse and bilateral white matter involvement is common in patients with *GRN* mutations. The presence of white matter lesions is not surprising since expression of PGRN, not only in neurons but also in activated microglia, in astrocytes and oligodendroglia, has been previously ascertained (Ahmed et al., 2010). As a consequence, cortical and subcortical loop are both implicated in the disruption of intrinsic brain networks in *GRN* mutations carriers.

Electroencephalography is a reliable and non-invasive tool for the study of brain networks in dementia. Relationship of the brain oscillations with intrinsic brain network like default mode network (DMN) have been extensively studied (Nishida et al., 2015). In particular, alpha and theta field potentials are deeply involved in tuning the large and local scale networks interactions in cognitive and psychiatric illnesses (Koenig et al., 2001; Tenke et al., 2011). Previous EEG studies have demonstrated peculiar modifications in brain oscillations in patients with MCI due to AD as compared to non-AD converters (Moretti et al., 2011a). Moreover, these changes in brain oscillations have been correlated with temporo-parietal and hippocampal atrophy (Moretti et al., 2007a, 2008a, 2009a,b, 2011a, 2012) as well as to white matter lesions (Moretti et al., 2007b, 2008b). In the present explorative study, we test the working hypothesis that changes in EEG oscillations could specifically detect different stages of FTLD, namely MCI-FTD versus overt FTD, as well as differences between *GRN* mutation carriers versus non- carriers. The search for new biomarkers is of great importance for an early diagnosis and for monitoring the effectiveness of new therapies.

## Materials and Methods

### Subjects

#### Diagnostic Criteria

Clinical diagnosis of FTLD and LBD were made according to international guidelines (McKeith et al., 1996; Neary et al., 1998) as well as more recent revised criteria for the diagnosis of frontotemporal dementia (Gorno-Tempini et al., 2011; Rascovsky et al., 2011). MCI-FTD patients were recruited with mini mental state examination (MMSE) score higher than 24/30. Eleven patients with MCI-FTD, eight FTLD patients carrying GRN mutations, and 20 FTLD patients not carrying *GRN* mutations were included in the present study (**Table 1**). The diagnosis of the enrolled subjects is reported in **Table 2**. All patients underwent a series of standardized diagnostic and severity instruments, including: the MMSE (Folstein et al., 1975), the Clinical Dementia Rating Scale (CDRS; Hughes et al., 1982), the Hachinski Ischemic Scale (HIS; Rosen et al., 1980), the Instrumental and Basic Activities of Daily Living (IADL, BADL; Lawton and Brody, 1969) and a comprehensive neuropsychological battery (Lezak et al., 2004). The patients were recruited only with apparently primary cognitive deficits excluding psychic comorbid conditions like anxiety, depression, psychosis etc., or physical comorbidities like hypothiroidism, uncontrolled diabetes, vitamin B12, and folate deficiency, uncontrolled heart disease or hypertension, drug addiction, or alcohol abuse. Moreover, none of the patients was taking any drugs that might affect the EEG, namely psychoactive drugs, including acetylcholinesterase inhibitors or other drugs enhancing brain cognitive functions. In addition, patients underwent diagnostic neuroimaging procedures (magnetic resonance imaging, MRI), and laboratory testing to rule out other causes of cognitive impairment. In particular, MRI was able to exclude patients with major cerebrovascular diseases or other diseases (like tumors) that might influence EEG frequency rhythms. On the whole, 13 patients with primary progressive non-fluent aphasia (PNFA), 12 patients with the behavioral variant of FTD (FTD-bv), one patient with the semantic variant of FTD (FTD-sv), and one patient with Lewy Body Disease (LBD) were recruited. Demographic and clinical features of the sample in study are reported in **Table 2**. All experimental protocols had been approved by the local ethics committee of Scientific Institute for Research and Care (IRCCS) of Alzheimer’s and psychiatric diseases ‘Fatebenefratelli’ in Brescia, Italy (Protocol numbers: 26/2014; 50/2015). Written informed consent was obtained from all participants or their caregivers, according to the Code of Ethics of the World Medical Association (Declaration of Helsinki).

**Table 1:** ANOVA results of demographic variable (age, education) MMSE score, and of PGRN dosage, high alpha, and theta EEG power of the study sample.

	GRN positive	GRN negative	MCI FTD	*p*
Number of subjects	8	20	11	–
Age, years	65,7 ± 9,3	67,2 ± 9,2	64,8 ± 4,2	0,07
Education, years	7,4 ± 2,8	7,6 ± 4,2	7,6 ± 3,1	0,9
MMSE	21,3 ± 5,6	20,5 ± 4,3	26,2 ± 5,1	0,11
PGRN dosage	33,6 ± 18,5	142,4 ± 72,1	n.a.	0.001
High alpha EEG power	15,2 ± 3,5	10,6 ± 3,2	8,7 ± 2,8	0,003
Theta EEG power	13,8 ± 5,1	21,1 ± 2,1	4,3 ± 8,3	0,002


**Table 2:** Clinical diagnosis of the patients recuited in the study.

DIAGNOSIS	PGRN positive	PGRN negative
FTD PPA	4	9
FTDbv	2	10
FTD	1	1
LBD	1	


*FTD, fronto-temporale dementia; PPA, primary progressive aphasia; bv, behavioral variant; LBD, Lewy body dementia*

### EEG Recordings

The EEG activity was recorded continuously from 19 sites by using electrodes set in an elastic cap (Electro-Cap International, Inc. Eaton, OH, USA) and positioned according to the 10–20 international system (Fp1, Fp2, F7, F3, Fz, F4, F8, T3, C3, Cz, C4, T4, T5, P3, Pz, P4, T6, O1, and O2). All recordings were obtained in the morning with subjects resting comfortably. The patients were instructed to sit with closed eyes and relax and vigilance was continuously monitored in order to avoid drowsiness by an operator. The ground electrode was placed in front of Fz. The left and right mastoids served as the reference points for all electrodes. The recordings were used off-line to re-reference the scalp recordings to the common average. Re-referencing was done prior to the EEG artifact detection and analysis. Data were recorded with a band-pass filter of 0.3–70 Hz and digitized at a sampling rate of 250 Hz (BrainAmp, BrainProducts, Munich, Germany). Electrode-skin impedance was set below 5 kΩ. Horizontal and vertical eye movements were detected by electrooculogram (EOG). The recording lasted 5 min with the subjects’ eyes closed. EEG data were then analyzed and fragmented off-line in consecutive epochs of 2 s with a frequency resolution of 0.5 Hz. The average number of epochs analyzed was 140, ranging from 130 to 150. The epochs with ocular, muscular, and other types of artifacts were discarded by two skilled electroencephalographists. The spectral power we obtained is an estimation of a spectrum collapsed all over the scalp electrodes. In this way, the eventual contribution of the muscular or other artifacts is strongly reduced. Moreover, two skilled electroencephalographists checked separately the EEG traces (Moretti et al., 2003, 2004).

### Analysis of Frequency Bands

A digital FFT-based power spectrum analysis (Welch technique, Hanning windowing function, no phase shift) computed the power density of EEG rhythms (ranging from 2 to 45 Hz) with a 0.5 Hz frequency resolution. The frequency bands range was computed on fixed limit as follows: (1) delta, 1–3 Hz; (2) theta, 4–7 Hz; (3) alpha 1 ore low alpha, 8–10,5 Hz; (4) alpha 2 ore high alpha, 10.5–14 Hz. These frequencies were computed on the power spectra averaged across all recording electrodes. This “collapsed spectrum method,” being a normalized scalp spectrum, determined a global field power (Moretti et al., 2003, 2004).

### Genetic Analysis

*GRN* gene was analyzed in patients with FTLD and LBD by direct sequencing of all exonic and flanking intronic regions as previously described (Benussi et al., 2008). Plasma PGRN levels were measured in duplicate using an ELISA kit (Human PGRN ELISA Kit, AdipoGen Inc., Seoul, Korea) as previously reported (Ghidoni et al., 2012b). *GRN* positive carriers belongs to different families. This choice has been made to avoid that EEG patterns could be due to familial relationships. **Tables 2** and **3** shows the clinical diagnosis and related genetic mutations of *GRN* positive carriers.

**Table 3:** Genetic mutations identified in the GRN mutations carriers group and related clinical diagnosis.

	PGRN identified mutations	Diagnosis
1	PGRN Leu271LeufsX10	FTDsv
2	PGRN Leu271LeufsX10 (g.1977_1980delCACT)	FTD-PPA
3	PGRN Leu271LeufsX10	FTD-PPA
4	PGRN Leu271LeufsX10	FTD-PPA
5	PGRN Thr276SerfsX7	FTD-PPA
6	PGRN Leu271LeufsX10	FTDbv
7	PGRN Leu271LeufsX10	FTDbv
8	PGRN Leu 271LeufsX10	LBD


*FTDsv, fronto-temporal dementia semantic variant; FTDbv, fronto-temporal dementia behavioral variant; FTD-PPA, fronto-temporal dementia primary progressive aphasia; LBD, Lewy, body disease.*

### Statistical Analysis

One-way analysis of variance (ANOVA) has been performed to compute the differences between groups (independent variables) in sociodemographic, MMSE score, PGRN dosage, and EEG oscillations (dependent variables) and neuropsychological tests. Age, education, and MMSE score have been used as covariates in ANOVA of brain rhythms to avoid confounding factors.

## Results

**Table 1** shows ANOVA significant results. As expected, the PGRN dosage was significantly different, with an impressive decrease in *GRN* null mutation carrier patients [*F*(19,77), df = 1, *p* < 0.001], confirming previous results (Ghidoni et al., 2012b). As about the EEG rhythms, results show: 1) a significant increase of high alpha power in *GRN* positive mutations carriers [*F*(4,22), df = 2, *p* = 0.003]; (2) a significant increase of theta power in *GRN* negative patients [*F*(3,14), df = 2, *p* = 0.002).

ANOVA analysis showed a decrease of the theta and alpha spectral power in MCI-FTD as compared to both PGRN positive and negative FTD patients. Given that the difference between PGRN positive and negative FTD patients were limited to theta and alpha power spectra, other frequency bands were not considered in the analysis.

The only significant results about neuropsychological battery were obtained in Token test, fluency for letters and working memory test. The results showed a general better performance in MCI-FTD patients as compared to both negative and positive PGRN mutations carriers (*p* < 0.01). No significant differences resulted between the comparison between negative and positive mutations PGRN carriers.

## Discussion

Our results show that FTLD patients carrying GRN mutations have different changes of EEG rhythms when compared to patients negative for GRN mutations. Specifically, GRN mutations carriers demonstrate an increment of the high alpha and a decreased of the theta power spectra. In this view, the presence of PGRN, which is a trophic factor for the nervous system, would allow a reorganization of the neural networks in a compensatory or adaptive way: accordingly, it has been reported that PGRN promotes neuronal survival and neurite outgrowth in cultured neurons (Van Damme et al., 2008) Likewise, under stress conditions it has been exhibited that PGRN improves neuronal survival (Kessenbrock et al., 2008). Data from GRN knockout experimental models suggest that PGRN deficiency leads to reduced synaptic connectivity and impaired plasticity, which may be contributing factors to FTLD pathology in human patients (Tapia et al., 2011; Xu et al., 2011). On the other hand, the white matter impairment in *GRN* mutation carriers has been directly proved in a recent study (Caroppo et al., 2014). In this study, four patients carrying *GRN* mutation were investigated and in all of them mostly confluent white matter lesions, affecting the periventricular subcortical white matter and U-fibers, were found mainly in the frontal and parietal lobes. This trophic activity could be shown by the maintenance of theta brain oscillations. Of note, fronto-parietal network are well recognized potential generators of theta oscillations (Beck et al., 2008; Ghetti et al., 2008; Whitwell et al., 2009), traveling on long pathway bundles of the white matter of fronto-parietal lobes and related subcortical structures like brainstem, and corpus callosum systems (Rohrer et al., 2010a,b). In particular, a recent study investigating the cortical generators of theta brain rhythm, through intracranial electrode recordings, have found that the higher percentage of cortical gated theta oscillations is situated in parieto-occipital regions (Raghavachari et al., 2006). So, a possible explanation of the spared maintenance of theta power in FTLD patients negative for GRN mutations as compared to GRN mutations carriers could be related to the relative integrity of parieto-occipital areas, embedded in the DMN.

Not surprisingly, in case of PGRN deficiency, the high alpha frequency spectral power increments. The alpha oscillation is the rhythm that mirrors the brain electrical signal of the parietal-occipital default system, profoundly connoted with higher cognitive abilities (Klimesch, 2012; Moretti, 2015a). Recent studies have shown that mutations in GRN are correlated with atrophy of the parietal lobes (Pickering-Brown et al., 2008; Seeley, 2008; Van Swieten and Heutink, 2008). In particular, a recent study investigating brain network connectivity with functional resting state MRI (Premi et al., 2014) utilizing three measures of both local and global connectivity (the regional homogeneity, the fractional amplitude of low frequency fluctuation and the degree centrality) has shown that parietal lobes are affected very early in *GRN* mutation carriers, so that the notion of fronto-parietal dementia for PGRN related disease should be considered. The parietal lobes are well-recognized core regions of the DMN (Sala-Llonch et al., 2015). Previous studies have demonstrated that high alpha rhythm synchronization (or increase) is a marker of loss of function in the default system, as exhibited in patients with prodromal or overt AD (Moretti et al., 2011b, 2013a,b, 2014a,b; Moretti, 2015b,c,d). The increase of high alpha oscillation in FTD patients, characterized by the loss of PGRN, confirms the reliability of this biomarker as a sign of DMN impairment. Moreover, the lack of PGRN is confirmed to be connected to the disruptive of the default system, determining real reverberations on cognitive capacity.

As expected, MCI-FTD have better cognitive performance, especially in frontal function assessment tests, as compare to overt FTD patients both carriers and non-carriers of GRN mutation. Moreover, in MCI-FTD the lower spectral power of theta and alpha power could be explained with an initial stage of the disease in which EEG changes are not still clear. A possible fingerprint of this stage could be the ratio between alpha and theta power spectra. The progression of disease could modify the EEG oscillations, as observed in overt FTD and more strongly in GRN mutation positive patients. Anyway, future studies will clarify if the presence or absence of PGRN mutations also in MCI-FTD could unveil more peculiar brain oscillations changes.

### Study Limitations

It should be remarked a main limitation of the study that is the small sample size. We want to highlight that it should be considered the exploratory nature of the study. In this view, the choice criteria and statistical outcomes are sufficiently robust to be confident in the overall reliability of the results. In particular, the straight difference between MCI-FTD and overt FTD patients confirm that results are not due to mere random chance results. Despite this limitation, it provides important results for future studies. Up to date, for the first time in this paper, the particular aspect of a neurophysiological EEG biomarker in GRN mutation carriers and non-carriers have been investigated. Most important, the detection of a neuropshysiological biomarker, related to functional, and structural changes, could open new windows on integrated research strategy about the molecular nexopathies shedding light on the way through which different proteinopathies could differently affect neural networks (Warren et al., 2013). Finally, the finding of a new biomarker could be helpful for early diagnosis, to monitor the progression of disease and to test disease-modifying drugs. Anyway, we are well aware of the need to correlate the results with a wider morphological and neuropathological characterization as well as with other risk factors.

## Conclusion

GRN mutations affect brain oscillatory activity. A better understanding of the complexity of PGRN biology in the brain will help guide the development of PGRN-modulating therapies for neurodegenerative disease. Further studies with a larger number of subjects are needed to confirm the present results.

## Author Contributions

DVM: Substantial contributions to the conception or design of the work. LB: substantial contribution for the acquisition, analysis, or interpretation of data for the work; and drafting the work. SF: substantial contribution for the acquisition, analysis, or interpretation of data for the work; AND drafting the work. MC: substantial contribution for the acquisition, analysis, or interpretation of data for the work; and drafting the work. GB: substantial contribution for the acquisition, analysis, or interpretation of data for the work; and drafting the work. RG: substantial contribution for the acquisition, analysis, or interpretation of data for the work; and drafting the work.

## Conflict of Interest Statement

The authors declare that the research was conducted in the absence of any commercial or financial relationships that could be construed as a potential conflict of interest.
